# Critical Assessment of Optimal Timing to Initiate Post-Stroke Rehabilitation

**DOI:** 10.26502/aimr.0258

**Published:** 2026-09-05

**Authors:** Sumanjali Reddy Kanmantha Reddy, Marcel P. Fraix, Devendra K. Agrawal

**Affiliations:** 1 Department of Translational Research, College of Osteopathic Medicine of the Pacific, Western University of Health Sciences, Pomona, California 91766 USA; 2 Physical Medicine and Rehabilitation, College of Osteopathic Medicine of the Pacific, Western University of Health Sciences, Pomona, California 91766 USA.

**Keywords:** Acute ischemic stroke, Acute hemorrhagic stroke, AVERT trial, Early Mobilization, Early rehabilitation, Early versus Delayed Stroke Rehabilitation, Neuroplasticity, Post-stroke rehabilitation

## Abstract

Early rehabilitation following stroke is widely advocated to prevent complications associated with immobility and promote neuroplasticity. However, the optimal timing of rehabilitation remains unclear, as clinical evidence regarding very early mobilization is inconsistent with its biological rationale. This review examines the biological rationale for early post-stroke rehabilitation, compares outcomes associated with early versus delayed rehabilitation, and evaluates factors that influence the optimal timing of post-stroke rehabilitation. Early rehabilitation may reduce immobility-related complications, cardiovascular deconditioning, and muscle atrophy, while promoting neuroplasticity during a narrow window in early stroke recovery. However, these biological benefits do not consistently translate into improved clinical outcomes. The AVERT trial demonstrated that high-dose, very early mobilization initiated within 24 hours of stroke onset can decrease the chance of favorable functional outcomes, highlighting the role of rehabilitation intensity in addition to timing. In contrast, other studies have demonstrated that earlier mobilization may improve activities of daily living (ADLs), functional independence, and hospital length of stay. The discrepancy in these findings suggests that post-stroke rehabilitation should be individualized according to stroke type, severity, hemodynamic and neurological stability, and patient-specific functional and psychosocial factors. Further research is needed to determine the optimal timing, intensity, and frequency of rehabilitation for different stroke populations. Personalized rehabilitation interventions may help maximize functional recovery, minimize harm, and improve overall quality of life.

## Introduction

Rehabilitation is a fundamental component of recovery following injury and illness. It is a patient-centered, multidisciplinary process with the goals of optimizing function, promoting independence, and reducing disability in individuals with health conditions [1–5]. Rehabilitation is utilized across numerous conditions, including neurologic conditions (e.g., stroke, traumatic brain injury, spinal cord injury), musculoskeletal trauma (e.g., anterior cruciate ligament tears, hip and knee arthroplasty, fractures), cardiopulmonary conditions, and critical illness [2,6–15].

Stroke is a severe neurological condition that continues to be a leading cause of adult mortality and disability. According to the American Stroke Association, “stroke occurs when a blood vessel that carries oxygen and nutrients to the brain is either blocked by a clot or bursts (or ruptures). When that happens, part of the brain cannot get the blood (and oxygen) it needs, so it and brain cells die” [16–24]. Strokes due to clots are known as ischemic, whereas strokes due to blood vessel burst/rupture are known as hemorrhagic. Acute ischemic stroke is the most common type of stroke, making up approximately 87% of all strokes, due to a thrombotic or embolic occlusion that blocks blood flow to the brain. Timely recognition and prompt management is crucial to reduce long-term disability and improve functional outcomes [25–31]. Therefore, rehabilitation is a critical component of post-stroke recovery.

While advances in care have reduced stroke mortality, the overall burden of stroke-related disability and long-term consequences continue to rise due to population growth and aging [32]. However, uncertainty still exists about the optimal timing of rehabilitation initiation following stroke. Although early mobilization can enhance functional recovery, evidence from the AVERT (A Very Early Rehabilitation Trial after stroke) trial, in which patients received very early rehabilitation within 24 hours of a stroke, demonstrated reduced odds of favorable outcomes compared to standard stroke care [33]. Therefore, this literature review evaluates the current evidence regarding the optimal time to begin rehabilitation interventions after acute ischemic and hemorrhagic stroke to determine how timing can influence overall functional outcomes.

## Methods

To identify relevant studies and findings for this literature review, a comprehensive literature search was conducted across multiple databases, including PubMed, ScienceDirect, and Web of Science, enabling the inclusion of high-quality research articles. Key search terms included: “acute ischemic stroke”, “acute hemorrhagic stroke”, “rehabilitation timing”, “early mobilization”, “delayed rehabilitation”, “multidisciplinary approach”, and “post-stroke rehabilitation”. We included studies published in English published from 2005 to current time and those which focused on acute ischemic and hemorrhagic stroke rehabilitation in adults over age 18. Non-English publications, studies that focused on pediatric stroke and non-ischemic and non-hemorrhagic stroke, and studies with a lack of sufficient data on functional outcomes were excluded. Data analysis of studies relied on patient demographics, rehabilitation interventions, biological rationale, timing of rehabilitation, and functional outcome measures.

### Biological Rationale for Early Rehabilitation Timing

**Figure 1:**
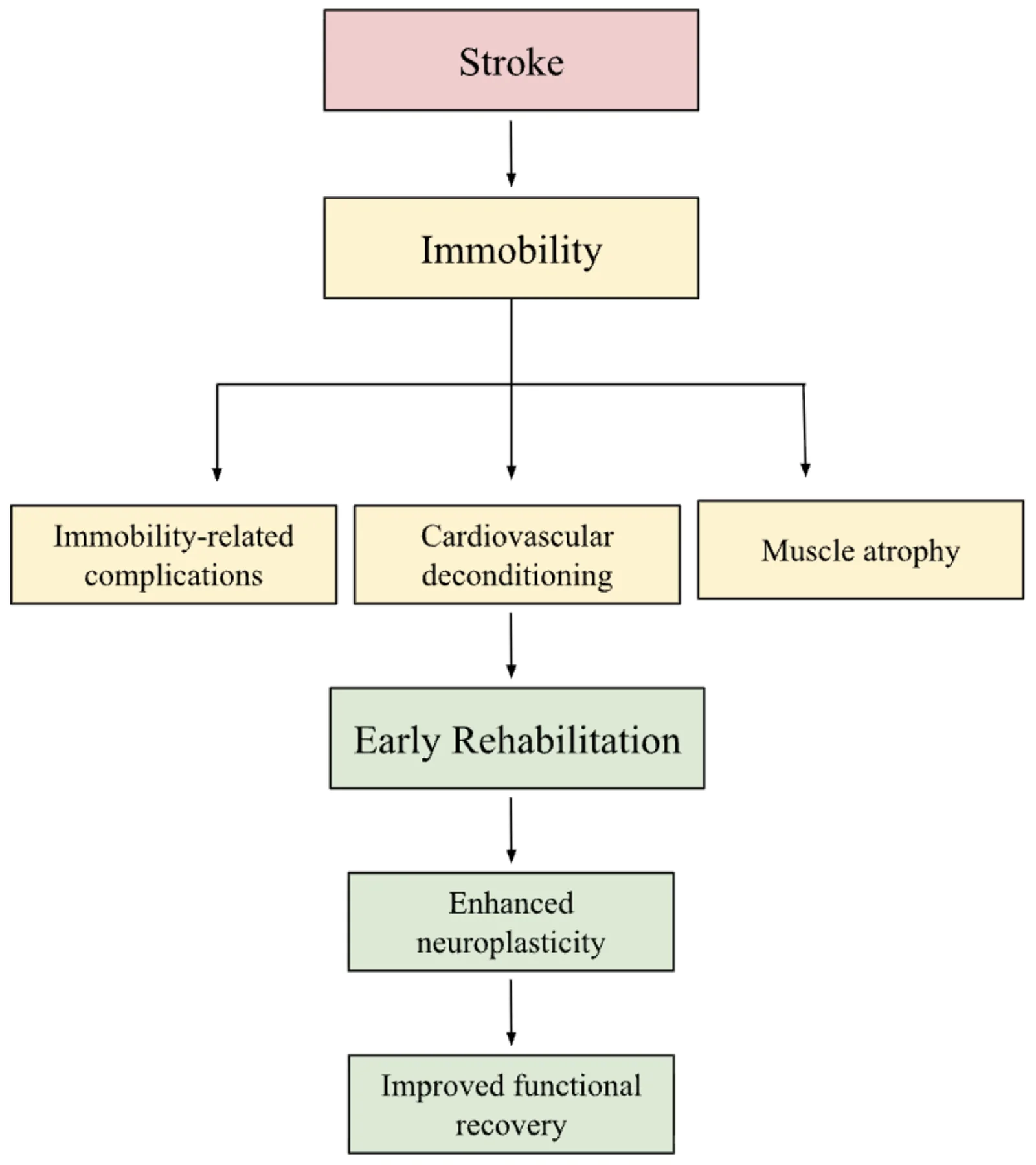
Biological Rationale for Early Rehabilitation Following Stroke. Stroke-related immobility can contribute to complications, cardiovascular deconditioning, and muscle atrophy. Early rehabilitation may mitigate these adverse effects while enhancing neuroplasticity, potentially supporting functional recovery.

Stroke can result in a wide range of long-term consequences, including aphasia, paresis, paralysis, gait impairment, dysphagia, language disorders, and depression. Post-stroke rehabilitation focuses on providing individualized therapy to promote structural and functional reorganization of the brain [34,35]. Treatment is multidisciplinary and aims to optimize the care pathway through collaboration among neurologists, physiatrists, neuropsychologists, and rehabilitation therapists such as physiotherapists, occupational therapists, and speech therapists [34]. As a key component of recovery, rehabilitation has been shown to improve functional outcomes and reduce long-term disability in patients recovering from stroke. Therefore, current stroke rehabilitation guidelines recommend early rehabilitation, as it promotes neuroplasticity and improves long-term functional independence [36]. The biological rationale supporting early post-stroke rehabilitation centers on preventing immobility-related complications, mitigating adverse effects on the musculoskeletal and cardiovascular systems, and promoting recovery during the early period of heightened neuroplasticity (Figure 1).

The main rationale for beginning early mobilization is to prevent complications related to immobility, including infections, deep vein thromboembolism, and falls. Additionally, physical inactivity following stroke can contribute to secondary complications such as reduced cardiovascular fitness and muscle atrophy [37]. A study assessing the effect of early stroke rehabilitation demonstrated significant improvement in cardiovascular fitness, with peak oxygen uptake (VO2peak) increasing by 24.8% following early stroke rehabilitation. The increase in peak oxygen uptake was also associated with greater functional improvement, emphasizing the role of early rehabilitation on reducing cardiovascular deconditioning [38,39]. Following stroke, patients can develop sarcopenia, defined as progressive loss of muscle mass, due to various factors. Prolonged periods of disuse lead to a disproportionate increase in proteolysis compared to protein synthesis, as well as a transformation in muscle fiber composition from type I (slow-twitch) fibers, which are more resistant to fatigue, to type II (fast-twitch fibers), which fatigue more quickly. As early as four hours after a stroke, there is a decrease in the number of motor units in the affected limb due to disruption of upper motor neuron pathways, contributing to muscle wasting [40]. In a study evaluating the level of C-terminal Agrin Fragment (CAF), a biomarker of sarcopenia, after acute stroke during early rehabilitation, results demonstrated decreased levels of CAF after rehabilitation training, suggesting the role of early rehabilitation in the prevention of abnormal muscle metabolism [41,42].

Evidence also suggests that the early post-stroke period represents a short-lived critical window of enhanced neuroplasticity. During this period, genes responsible for axonal growth, dendritic spine formation, and synaptogenesis are upregulated in peri-infarct areas, suggesting that early initiation of rehabilitation may maximize neuroplasticity, promote recruitment of surviving neuronal networks, and enhance motor recovery [36,37]. It was shown that during rehabilitation, there is an increased release of neurotrophic growth factors such as vascular endothelial growth factor (VEGF), which stimulates angiogenesis around the lesion, and brain-derived endothelial growth factor (BDNF), which promotes neurogenesis and neuroprotection [39]. Importantly, BDNF has emerged as a key mediator of neuroplasticity, specifically in improving motor function. Evidence suggests that aerobic exercise is valuable in improving brain function through upregulation of BDNF gene expression, which enhances neuroplasticity by facilitating long-term potentiation (LTP). LTP is a persistent strengthening between neurons when they are repeatedly activated together, improving not only post-stroke cognitive function but also post-stroke mobility, balance, and motor function [43]. Early rehabilitation involving aerobic exercise has also been found to improve post-stroke cognitive function through increased cerebral blood flow, providing metabolic support for neuroplasticity [44].

Overall, the biological rationale for early rehabilitation after stroke extends beyond preventing complications of immobility. By mitigating cardiovascular deconditioning and muscle atrophy while promoting neuroplasticity, early rehabilitation may help preserve physical function, optimize neurological recovery, and ultimately improve long-term functional outcomes.

### Comparative Analysis of Early Versus Delayed Rehabilitation

Although the biological mechanisms underlying early rehabilitation suggest that it may promote functional recovery, clinical evidence regarding optimal timing of rehabilitation is inconsistent. While early mobilization can promote neuroplasticity and mitigate complications associated with immobility, these biological benefits do not necessarily result in improved functional outcomes when mobilization is initiated too early or delivered at high intensity. Therefore, clinical studies are needed to determine how timing and intensity of rehabilitation can be optimized to translate biological benefits into meaningful improvements in patient recovery.

A Very Early Rehabilitation Trial (AVERT) is a major international and randomized controlled trial that tested the safety, feasibility, and effectiveness of very early mobilization (VEM) within 24 hours of stroke onset [45]. In this trial, patients with ischemic or hemorrhagic stroke were randomized to receive either standard stroke-unit care alone or very early mobilization in addition to standard care. Very early mobilization began within 24 hours of stroke onset and consisted of out-bed sitting, standing, and walking activities [46]. The primary outcome measure was survival without significant disability at 3 months after stroke, defined as a modified Rankin scale (mRS) score of 0-2. Secondary outcomes included time required to walk 50 m independently and occurrence of serious adverse events at 3 months after stroke, as well as quality of life and cost effectiveness at 12 months [46,47].

The phase II study of AVERT, which evaluated the safety and feasibility of a very early rehabilitation protocol, concluded that VEM could be safely implemented and was feasible in the acute stroke setting [48]. However, findings from the subsequent phase III trial reported that high-dose, very early mobilization within 24 hours of stroke onset can significantly lower the chance of favorable functional outcomes at 3 months compared with standard care. Additionally, no statistically significant differences were observed between groups in secondary outcomes at 3 months and quality of life at 12 months [46]. These findings suggest that while early mobilization is feasible and can be safely implemented, initiating high-dose mobilization within the first 24 hours after stroke may adversely affect functional recovery. Most importantly, the AVERT findings highlight the importance of considering not only the timing of rehabilitation initiation but also the intensity and frequency of mobilization in post-stroke care.

Findings from other studies provide additional evidence regarding the effects of VEM may vary depending on the outcomes assessed and the rehabilitation protocol used. A study comparing VEM initiated within 48 hours of stroke onset with usual care, in which time to first mobilization was initiated later, found that patients receiving VEM had higher mean activities of daily living (ADL) scores, as measured by the Barthel Index, a measure of functional independence, and a shorter mean length of hospital stays compared with patients receiving usual care. However, VEM did not increase the proportion of patients who survived or achieved a favorable functional recovery after their stroke [49]. Similarly, Momosaki et al. analyzed the association between very early versus delayed rehabilitation in patients with acute ischemic stroke who received intravenous recombinant tissue plasminogen activator and found that very early rehabilitation was significantly associated with a higher proportion of patients achieving functional independence [50]. Another study evaluating very early mobilization, beginning within 24 hours of stroke onset, consisting of 5-30 minutes of activity at least twice daily for seven days, found that patients in the intervention group demonstrated significantly greater improvement in Barthel Index scores, as well as functional status at discharge and at three months follow up compared to the standard care group [51].

Taken together, the evidence suggests that the effects of earlier mobilization may be outcome specific, with some studies demonstrating improvements in functional independence, activities of daily living, and hospital length of stay, while others have not demonstrated improvements in outcomes such as favorable functional recovery. Importantly, the findings from AVERT further emphasize that the optimal timing of post-stroke rehabilitation must be considered alongside intensity and frequency of therapy.

### Factors Influencing Optimal Rehabilitation Timing

**Figure 2:**
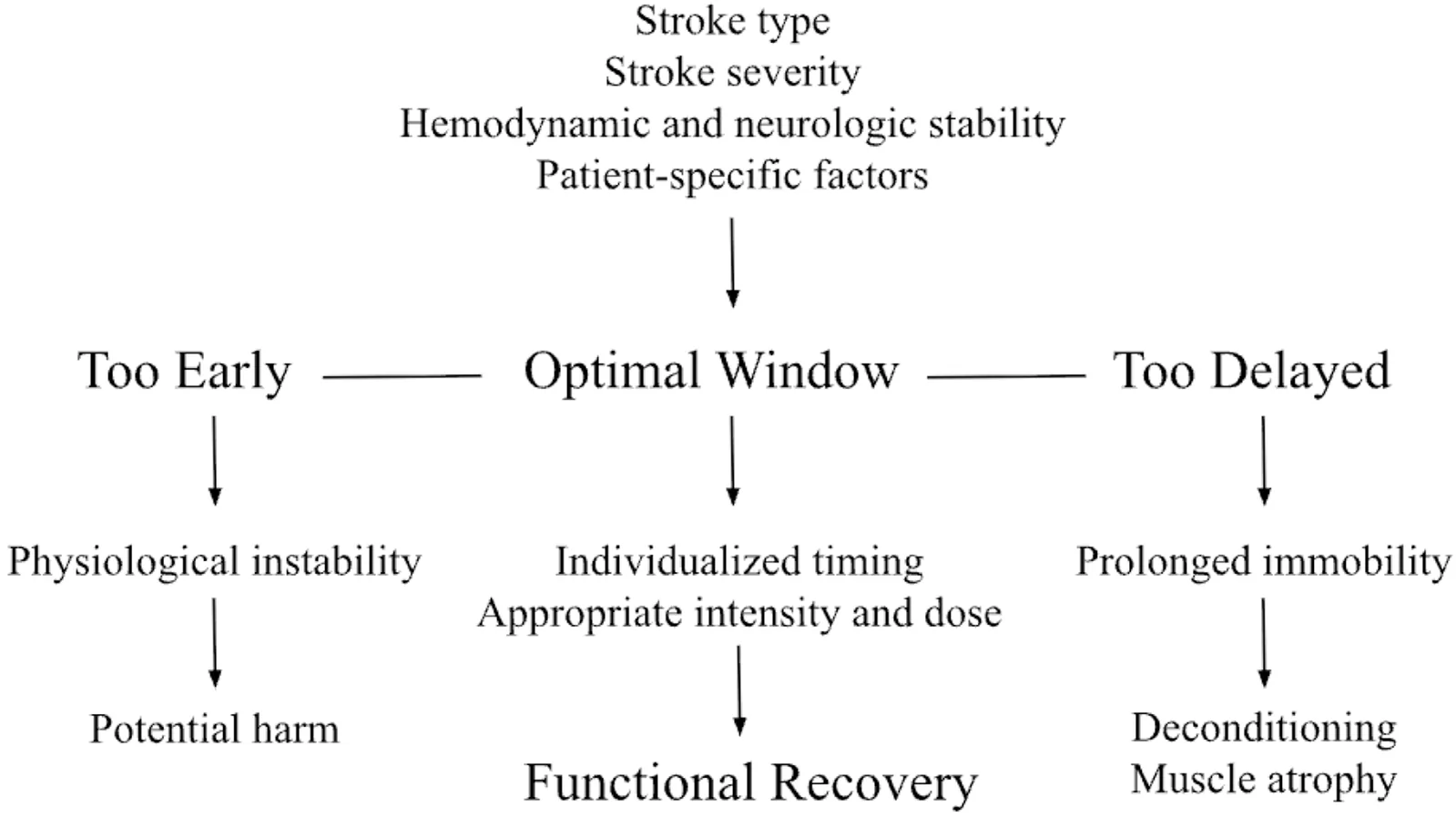
Factors Influencing Optimal Timing of Post-Stroke Rehabilitation. The optimal timing of rehabilitation represents an individualized window between the potential risks of initiating mobilization before achieving medical and neurological stability and the adverse effects of prolonged immobility. Stroke type, severity, hemodynamic and neurological stability, and patient-specific factors should be considered when tailoring the timing and intensity of rehabilitation to the patient’s clinical status.

The optimal timing of post-stroke rehabilitation should be individualized based on clinical and patient-specific factors (Figure 2). It is important to consider stroke type (ischemic versus hemorrhagic), stroke severity, hemodynamic stability, and psychosocial factors.

Stroke type is an important consideration when determining timing of post-stroke rehabilitation, as the acute management differs between ischemic and hemorrhagic stroke. The 2022 American Heart Association/American Stroke Association guidelines suggest that implementation of rehabilitation activities may be considered 24 to 48 hours after moderate intracerebral hemorrhage (ICH). However early aggressive mobilization within the first 24 hours after ICH may increase risk of mortality [52]. The cautious approach reflects the importance of medical and neurological stabilization before initiating intensive rehabilitation. Naito et al. evaluated the inhibitors of early mobilization in 322 patients with ICH and reported that impaired consciousness, greater hematoma volume and growth, surgery, and markers of acute illness at admission were associated with delayed mobilization. Thus, rehabilitation following ICH should be initiated after sufficient medical and neurological stabilization [53]. Additionally, stroke severity should be considered when determining rehabilitation timing following ischemic and hemorrhagic stroke, as greater neurological impairment may limit a patient’s ability to safely participate in early mobilization. In contrast, evidence from ischemic stroke populations supports potential benefits of early mobilization. A randomized controlled trial evaluating the difference between starting physical rehabilitation therapy between 24 and 48 hours and 72 and 96 hours following onset of ischemic stroke found that early rehabilitation therapy between 24 and 48 hours had more favorable functional outcomes [54]. These findings highlight the importance of tailoring timing of rehabilitation to stroke type and patient’s clinical status.

Hemodynamic stability is another important consideration when determining timing of rehabilitation. Early mobilization may be associated with fluctuations in arterial blood pressure. Yagi et al. [55] found that among patients with acute ischemic stroke after mechanical thrombectomy, prominent changes in blood pressure occurred more frequently during an early mobilization protocol, emphasizing the importance of monitoring hemodynamic responses during rehabilitation. This consideration is particularly relevant following ICH, in which blood pressure management is critical due to impaired cerebral autoregulation. Increased systolic blood pressure can disrupt the blood-brain barrier, resulting in vasogenic edema, hematoma expansion, and rebleeding [56]. Therefore, it is important to assess hemodynamic stability before initiating early mobilization and adjust the timing and intensity of rehabilitation according to the patient’s physiological status.

Lastly, psychosocial factors may influence a patient’s willingness and ability to participate in rehabilitation. Financial burden, depression, self-efficacy, social support, disability acceptance, and environmental factors have been associated with rehabilitation motivation and therapeutic outcomes. Therefore, integrating individualized motivational assessments into clinical practice and developing interdisciplinary interventions to address these motivational barriers may help improve engagement in rehabilitation and long-term patient outcomes [57,58].

### Stroke Rehabilitation Outcome Measures

Stroke rehabilitation outcomes can be measured using a variety of standardized measures, including the modified Rankin Scale (mRS), Barthel Index (BI), and Functional Independence Measure (FIM). The modified Rankin Scale (mRS) is a 7-level scale used to assess the degree of disability and functional dependence in daily activities following stroke, ranging from no symptoms to death. It has become one of the most used primary outcome measures in acute stroke trials [59,60]. The Barthel Index (BI) is a widely used measure of functional independence in activities of daily living following stroke. It assesses 10 domains, including bowel and bladder control, grooming, feeding, toilet use, transfers, mobility, dressing, stair climbing, and bathing. Scores range from 0 to 20, with lower scores indicating greater disability and dependence and higher scores indicating greater functional independence [61]. Lastly, Functional Independence Measure (FIM) is a tool used to measure a patient’s level of disability and functional independence. It consists of 18 items divided into 13 motor and 5 cognitive tasks. Motor domains can include self-care, sphincter control, locomotion, and transfers, while cognitive tasks include communication and social cognition. Each item is scored from 1 to 7, where 1 indicates total dependence and 7 indicates complete independence. Total scores range from 18 to 126, with higher scores indicating greater functional independence in activities of daily living [62]. Together, these standardized measures provide assessments of recovery following stroke.

## Conclusion

In conclusion, early rehabilitation following stroke is supported by a strong biological rationale, including the prevention of immobility-related complications, reduction of cardiovascular deconditioning and muscle atrophy, and promotion of neuroplasticity. However, clinical evidence suggests that earlier rehabilitation does not necessarily result in improved outcomes, particularly when mobilization is initiated too early or at a high intensity. Optimal rehabilitation timing should therefore be individualized according to stroke type, severity, hemodynamic and neurologic stability, and patient specific factors. Further research is needed to better define the timing, intensity, and frequency of rehabilitation that can maximize functional recovery while minimizing potential harm. Expanding our understanding of optimal rehabilitation timing can contribute to the development of more precise clinical guidelines and individualized rehabilitation strategies for post-stroke care, helping stroke survivors regain functional independence and achieve a higher quality of life.

## Key points and outstanding questions

- Early rehabilitation after stroke has a strong biological rationale due its potential to reduce immobility-related complications, cardiovascular deconditioning, and muscle atrophy while promoting neuroplasticity.
- Early rehabilitation during the narrow window of heightened neuroplasticity following stroke may promote cognitive and functional recovery.
- The biological benefits of early rehabilitation do not necessarily translate into improved clinical outcomes, as evidence regarding its effectiveness remains inconsistent.
- The AVERT trial demonstrated that high-dose, very early mobilization within 24 hours may reduce likelihood of favorable functional outcomes at 3 months compared to standard care.
- Other clinical studies demonstrated benefits from early rehabilitation, including improvements in functional independence, activities of daily living, and decreased hospital length of stay, highlighting the role of intensity and frequency with early mobilization.
- Rehabilitation timing should be individualized based on stroke type, stroke severity, hemodynamic and neurological stability, and psychosocial factors.
- Can biomarkers help identify patients who will most likely benefit from early rehabilitation?
- What is the optimal intensity, frequency, and duration of rehabilitation during the early stages of stroke recovery to maximize functional outcomes and minimize harm?
